# Suicide among Arab-Americans

**DOI:** 10.1371/journal.pone.0014704

**Published:** 2011-02-17

**Authors:** Abdulrahman M. El-Sayed, Melissa Tracy, Peter Scarborough, Sandro Galea

**Affiliations:** 1 Department of Epidemiology, Columbia University, New York, New York, United States of America; 2 Department of Public Health, University of Oxford, Oxford, United Kingdom; 3 College of Physicians and Surgeons, Columbia University, New York, New York, United States of America; 4 Center for Social Epidemiology and Population Health, Department of Epidemiology, University of Michigan School of Public Health, Ann Arbor, Michigan, United States of America; RAND Corporation, United States of America

## Abstract

**Background:**

Arab-American (AA) populations in the US are exposed to discrimination and acculturative stress—two factors that have been associated with higher suicide risk. However, prior work suggests that socially oriented norms and behaviors, which characterize recent immigrant ethnic groups, may be protective against suicide risk. Here we explored suicide rates and their determinants among AAs in Michigan, the state with the largest proportion of AAs in the US.

**Methodology/Principal Findings:**

ICD-9/10 underlying cause of death codes were used to identify suicide deaths from among all deaths in Michigan between 1990 and 2007. Data from the 2000 U.S. Census were collected for population denominators. Age-adjusted suicide rates among AAs and non-ethnic whites were calculated by gender using the direct method of standardization. We also stratified by residence inside or outside of Wayne County (WC), the county with the largest AA population in the state. Suicide rates were 25.10 per 100,000 per year among men and 6.40 per 100,000 per year among women in Michigan from 1990 to 2007. AA men had a 51% lower suicide rate and AA women had a 33% lower rate than non-ethnic white men and women, respectively. The suicide rate among AA men in WC was 29% lower than in all other counties, while the rate among AA women in WC was 20% lower than in all other counties. Among non-ethnic whites, the suicide rate in WC was higher compared to all other counties among both men (12%) and women (16%).

**Conclusions/Significance:**

Suicide rates were higher among non-ethnic white men and women compared to AA men and women in both contexts. Arab ethnicity may protect against suicide in both sexes, but more so among men. Additionally, ethnic density may protect against suicide among Arab-Americans.

## Introduction

Little is known about the health of Arab-Americans (AAs) in the United States. A recent review of research into the health of AAs found only 34 studies in the peer-reviewed literature that empirically assessed health metrics among this group [1]. The health of AAs may differ systematically from other groups in the US for several reasons [1]: first, AAs are relatively recent immigrants to the US [2]; second, AAs share a set of cultural norms that may be particularly influenced by Islamic behavioral restrictions; and third, following sociopolitical events in recent times, including the terrorist attacks of September 11, 2001, and the subsequent wars in Iraq and Afghanistan, AAs have been increasingly marginalized from the general population [1], [3], [4].

Therefore, AA populations may be exposed to higher levels of discrimination and acculturative stress, which have both been shown to influence health outcomes among this group [1]. Several studies that have explored the mental health of AAs in the US have shown that discrimination may be an important determinant of adverse mental health. In a study among AAs in north central Florida [5], self-reported incidents of discrimination were associated with psychological distress and lower self-esteem. A recent study by Padela and Heisler [6] considered the relation between discrimination and mental health among a representative sample of AAs in metropolitan Detroit and found that self-reported experiences of discrimination were associated with higher levels of psychological distress [6].

Acculturation has also been shown to influence AA mental health. Among second generation and early immigrant AAs from 19 states and the District of Columbia, Amer and Hovey [7] showed that greater adherence to traditional Arab/religious family values and greater family dysfunction were independent risk factors for depression. A study by Ajrouch [8] assessed the relation between acculturation and psychological wellbeing among elderly AAs, and showed that immigrant status was associated with more frequent feelings of depression and lower life satisfaction. Wrobel and colleagues [9] similarly found that acculturative stress was a predictor of depression among a sample of AAs between 60–92 years old.

Conversely, however, research about suicide among ethnic minority groups in the US has consistently demonstrated that these populations may have lower risk for suicide than their white counterparts. Socially-oriented norms, such as communalism and a collective social orientation, affective expressiveness, strong family bonds, and positive ethnic group identity among ethnic minority groups have been cited as important mechanisms through which ethnic minorities may be protected against suicide [10]. Although, to our knowledge, there has been no published research about suicide among AAs, it is plausible that AAs, a recent and tight-knit minority population [11]–[13], may be protected against suicide by similar mechanisms.

Communal characteristics that may protect against suicide among recent immigrant groups in the US may be particularly strong in ethnic enclaves. Such enclaves, characterized by high ethnic density, maintain a cultural distinction from surrounding contexts [14] where ethnic inhabitants may be more likely to maintain many of the cultural features described above. Ethnic populations in ethnic enclaves have been shown be protected against diverse adverse outcomes, including adverse birth outcomes [15], [16], all-cause mortality [17], asthma [18], and psychopathology [19]. Of particular interest here is literature suggesting that ethnic residents of ethnic enclaves may be protected against suicide [20], [21]. For example, one study by Neeleman and Wessely demonstrated that high ethnic density was protective against suicide risk among ethnic minorities in London [20].

As little is yet known about suicide risk among this growing ethnic minority group, studies about the prevalence and determinants of suicide among the AA population are needed. Using data on all deaths between 1990 and 2007 in the US state of Michigan, the state with the highest density of AAs in the US, as well as Census 2000 population data, we explored age-adjusted suicide rates among the AA and non-ethnic (non-Arab and non-Hispanic) white populations. Given that ethnicity may influence suicide rates differently by ethnic density [20], [21], we stratified our analyses by residence inside and outside of Wayne County, MI, the county with the highest density of AAs in Michigan and the US, as well as home to 39% of the state's AAs, to understand the relations between Arab ethnicity, ethnic density, and suicide risk.

## Methods

### Data

We obtained data about all deaths among individuals aged 10 years and older in the state of Michigan between 1990 and 2007 from the death records of the Michigan Department of Community Health (MDCH). US Census 2000 population data, obtained using the American Factfinder Database [22] and the Integrated Public Use Microdata Series [23], were used to calculate population denominators (year 2000 data were used as denominators in all years).

Our primary outcome of interest was death by suicide. We used International Classification of Diseases (ICD) codes to determine deaths caused by intentional self-harm (suicide) or unaccounted injury. ICD-9 codes E950-E959 and E980-E989 were used from 1990 through 1998, and ICD-10 codes X60-X84 and Y10-Y34 were used from 1999 through 2007. It has been shown that there is no difference in the proportion of deaths attributed to suicide by coding edition [24].

Other covariates collected in mortality data included race, ethnicity, gender, and age. Only suicide among AAs and non-ethnic whites was considered, but this included over 80% of suicides in Michigan. Arab ethnicity was determined by next-of-kin-reported ancestry and white race was determined by next-of-kin-reported race in mortality data, while self-reported ancestry and race was used in population data.

Suicide rates were stratified by residence inside or outside of Wayne County, MI. With approximately 39% of Michigan's Arab-American population residing therein, Wayne County is home to one of the largest Arab-American communities in the US [25]. For example, it is estimated that one in three residents of the city of Dearborn, Wayne County's most Arab-dense city, is Arab-American [25]. Wayne county is also the largest county in Michigan, both by land area and population, and includes within its boundaries the City of Detroit. As compared to the rest of the State, Wayne County has a smaller white population (55.1% vs. 81.2%), a larger African-American population (40.5% vs. 14.2%), and a larger foreign-born population (6.7% vs. 5.3%) [26]. Residents of Wayne County are generally less educated (Proportion with bachelor's degree or higher, 17.2% vs. 21.8%) and lower income (Median household income, $42,463 vs. $48,606) than their counterparts in the rest of the state [26]. For purposes of this analysis, residence in Wayne County, MI was determined from next-of-kin-reported county of residence in mortality data.

In order to describe the underlying population of interest (Arab and non-Arab white Michigan residents aged 10 years and older), we collected information from the U.S. Census on marital status (married, separated, divorced, widowed, and never married); education (none, less than 11^th^ grade, general equivalency diploma (GED) or equivalent, and some college or greater); household income (negative, <$20,000, $20,001–$40,000, $40,001–$60,000, $60,001–$80,000, and greater than $80,000); gender (male vs. female); and age (10–19, 20–24, 25–44, 45–65, 65 and older).

This study was reviewed by the Institutional Review Board of the Michigan Department of Community Health and the Health Science Institutional Review Board of the University of Michigan.

### Analysis

First, we calculated univariate statistics stratified by ethnicity and gender to describe the population of interest. Second, we used bivariate chi-square tests, stratified by gender, to identify statistically significant distributions of covariates of interest by ethnicity (p<0.05). The Integrated Public Use Microdata Series [23] interface was used to carry out univariate statistics and bivariate chi-square tests of the population denominator data described above.

Third, we calculated age-adjusted suicide rates stratified by gender and ethnicity, as well as by gender, ethnicity, and residence inside or outside of Wayne County, MI. The year 2000 population standard was used for age adjustment, with all suicide rates standardized in five-year age categories. Fourth, we calculated the relative risk of suicide among AA men and women relative to their non-ethnic white counterparts, as well as among residents of Wayne County, MI relative to non-residents stratified by gender and ethnicity.

## Results

Table 1 shows univariate statistics and bivariate chi-square tests between demographic factors among AA and non-ethnic white Michigan residents in 2000, stratified by gender. Among both males and females, AAs were better educated (p<0.01), reported higher household incomes (p<0.01), and were older (p<0.01) than their non-ethnic white counterparts. Among males, AAs were more likely to be married (p<0.01) than non-Arab whites.

**Table 1:** Demographic characteristics of Arab-American and non-ethnic white males and females aged 10 and older in Michigan, 2000.

	Males			Females		
	Arab ethnicity	non-ethnic white		Arab ethnicity	non-ethnic white	
Total population (N)	15,749	3,425,585		15,941	3,586,494	
						
	%	%	p-value	%	%	p-value
Education			<0.01			<0.01
None	1.4	0.7		1.9	0.6	
<11 grade	23.8	27.0		25.0	25.0	
GED or equivalent	16.9	25.9		21.8	28.7	
Some college or greater	57.9	46.5		51.4	45.7	
Household income*			<0.01			<0.01
Negative	0.4	0.5		1.3	0.6	
<20,000	8.2	9.2		13.5	13.9	
20–40,000	16.2	20.3		17.9	21.5	
40–60,000	20.3	21.6		18.4	20.2	
60–80,000	16.0	17.3		13.0	15.7	
>80,000	39.0	31.0		35.8	28.1	
Marital status			0.01			0.07
Married	57.3	52.1		46.6	49.8	
Separated	1.8	2.7		2.2	2.5	
Divorced	5.5	8.3		9.8	10.0	
Widowed	1.6	2.4		10.6	9.8	
Single, never married	33.7	34.5		30.6	28.0	
Age			0.01			0.02
10–19	13.4	17.1		16.8	15.6	
20–24	8.0	7.6		5.8	6.9	
25–44	35.8	35.0		30.9	33.2	
45–64	26.4	27.3		24.8	26.6	
65+	16.4	13.0		21.7	17.7	

*in US dollars per year.

Table 2 shows age-adjusted suicide rates overall and by age group stratified by gender and ethnicity, as well as relative risks of suicide among AAs relative to non-ethnic whites by gender. The suicide rate overall was 25.10 per 100,000 per year among males and 6.40 per 100,000 per year among females. Among AAs, overall rates of suicide were lower among both males (12.43 in 100,000) and females (4.86 in 100,000) than among non-ethnic white males (25.27 in 100,000) and females (6.42 in 100,000). Among males, relative risk of suicide overall among AAs compared to non-ethnic whites was 0.49 while relative risk among females was 0.76. Similar relative risks were found across all age groups, except among elderly women, where AA women had 32% higher risk of suicide than their white counterparts.

**Table 2:** Age-adjusted suicide rates (per 100,000 per year) by ethnicity and gender, as well as relative risks of suicide comparing Arab ethnicity to non-ethnic whites aged 10 and older in Michigan, 1990–2007.

	Female				Male			
	Overall	Arab ethnicity	non-ethnic white	Relative risk	Overall	Arab ethnicity	non-ethnic white	Relative risk
Overall Suicide Rate	6.40	4.86	6.42	0.76	25.10	12.43	25.27	0.49
10–19	1.91	0.00	1.94	0.00	9.08	2.38	9.19	0.26
20–25	4.60	1.33	4.65	0.29	25.17	13.92	25.37	0.55
25–44	8.13	5.84	8.16	0.72	28.45	11.69	28.73	0.41
45–64	8.47	6.94	8.48	0.82	25.47	17.32	25.55	0.68
65+	4.80	6.31	4.79	1.32	34.91	16.35	35.01	0.47

Table 3 shows age-adjusted suicide rates overall and by age group among AAs stratified by gender and residence in Wayne County, MI, as well as relative risks of suicide among Wayne County, MI AA residents relative to AA non-residents. The suicide rate among male AAs in Wayne County, MI was 10.03 per 100,000 per year, and 4.36 per 100,000 per year among females. Outside of Wayne County, MI, suicide rates were 14.12 per 100,000 per year among AA males and 5.43 per 100,000 per year among AA females. The relative risk of suicide comparing Wayne County, MI residents to non-residents was 0.71 among males and 0.80 among females.

**Table 3:** Age-adjusted suicide rates (per 100,000 per year) inside and outside of Wayne county as well as relative risk of suicide in Wayne county relative to all other counties among Arab ethnicity males and females aged 10 and older in Michigan, 1990–2007.

	Female			Male		
	Wayne	All other	Relative risk	Wayne	All other	Relative risk
Overall Suicide Rate	4.36	5.43	0.80	10.03	14.12	0.71
10–19	0.00	0.00	–	4.56	0.00	–
20–25	0.00	2.73	0.00	14.87	12.85	1.16
25–44	4.3	7.11	0.60	8.60	14.46	0.59
45–64	6.92	7.07	0.98	15.06	19.05	0.79
65+	7.33	6.27	1.17	8.23	21.56	0.38

Table 4 shows age-adjusted suicide rates overall and by age group among non-ethnic whites stratified by gender and residence in Wayne County, MI, as well as relative risks of suicide among non-ethnic white Wayne County, MI residents relative to non-ethnic white non-residents. The suicide rate among non-ethnic whites in Wayne County, MI was 27.84 per 100,000 per year among males, and 7.32 per 100,000 per year among females. Outside of Wayne County, MI, suicide rates were 24.88 per 100,000 per year among males and 6.29 per 100,000 per year among females. Relative risk of suicide comparing Wayne County, MI residents to non-residents was 1.12 among males and 1.16 among females.

**Table 4:** Age-adjusted suicide rates (per 100,000 per year) inside and outside of Wayne county as well as relative risk of suicide in Wayne county relative to all other counties among non-ethnic white males and females aged 10 and older in Michigan, 1990–2007.

	Female			Male		
	Wayne	All other	Relative risk	Wayne	All other	Relative risk
Overall Suicide Rate	7.32	6.29	1.16	27.84	24.88	1.12
10–19	2.10	1.92	1.09	8.15	9.33	0.87
20–25	6.58	4.40	1.50	33.87	24.21	1.40
25–44	9.31	7.99	1.17	32.45	28.13	1.15
45–64	9.16	8.38	1.09	29.02	25.05	1.16
65+	5.81	4.62	1.26	34.31	35.16	0.98

## Discussion

In a study of all suicide deaths among AAs and non-ethnic whites in Michigan between 1990 and 2007 we found that suicide rates among AAs were lower than among non-ethnic whites across gender. We also found that while residence in Wayne County, MI was associated with higher rates of suicide among non-ethnic whites, it was associated with lower rate of suicide among AAs compared to residence in the rest of the state.

This is the first study, of which we are aware, that has considered suicide rates among AAs. While there is no previous data comparing suicide rates for AAs to non-ethnic whites, our findings are consistent with previous studies that have shown that whites have higher risk for suicide than other ethnic and racial minorities, including Hispanic-Americans [27], [28], African-Americans [29]–[31], and Asian-Americans [32].

Several factors have been suggested that may explain the lower rates of suicide among minority groups compared to non-ethnic whites, including stronger religious belief systems and spiritually-based coping, communalism and a collective social orientation, strong family bonds, affective expressiveness, and positive ethnic group identity among ethnic minority groups [10]. While there is no research, of which we are aware, that has analyzed these factors with regard to the etiology of AA suicide, it is plausible that many of these protective factors apply to AAs in the US, and therefore may explain lower risk for suicide among AAs relative to non-ethnic whites, as well.

Moreover, according to traditional interpretations of Islamic *shari'a* law, suicide is considered *haram* or forbidden, and families of suicide victims can face ostracism within the Muslim community [33], [34]. Both explicit religious restrictions against suicide among Muslim AAs, and the effect that Islamic restrictions may have on the communally-held view of suicide within broader Arab culture may also partially explain the lower risk of suicide among AAs relative to non-ethnic whites.

We also found that, despite higher suicide rates among non-ethnic whites in this context, AAs in Wayne County had lower suicide rates than their counterparts in other contexts. These findings support the extant literature about ethnic density and suicide among ethnic minority groups [20], [21], which has demonstrated that ethnic density may be protective against suicide and deliberate self-harm among ethnic minorities groups.

Our observations regarding ethnic density and AA suicide rates may best be understood within the broader framework of acculturation and health outcomes among ethnic minorities [16], [17], [35]–[38]. Ethnic enclaves maintain a cultural distinction from their surroundings [14]. Therefore, ethnic inhabitants of ethnic enclaves may be more likely to adhere to traditional cultural practices, and therefore be less acculturated, than their counterparts in other localities. If traditional cultural practices among ethnic minorities influence health, than health metrics among ethnic minorities may differ systematically by ethnic density because of cultural practice norms. Suggestive evidence comes from several studies that have demonstrated that community context influences health metrics among ethnic minority groups, suggesting that acculturation may mediate these differences [15]–[17].

As discussed above, ethnic minorities may be protected against suicide via communally-oriented cultural features, including communalism and a collective social orientation, strong family bonds, affective expressiveness, and positive ethnic group identity [10]. If ethnic inhabitants of ethnic enclaves, who may be less acculturated, may be more likely to adhere to such traditional cultural norms, then it is plausible that these mechanisms may operate more robustly in these contexts, explaining our finding that AAs in Wayne County, MI, home of one of the US's largest and most ethnically-dense Arab enclaves, had lower suicide rates than their counterparts outside of Wayne County, MI.

There are several limitations that should be considered when interpreting our findings. First, our observations come from only one US state. Therefore, they may not generalize to other contexts within the US or internationally. Second, while population suicide rates were stratified by ethnicity and gender and adjusted for age, our methods did not account for any other factors that may have confounded the relation between ethnicity and suicide. Third, we are limited by the accuracy of death certificate data collected from vital registry files and population statistics from Census 2000 data. Fourth, population data were taken only from the 2000 Census and therefore our analysis cannot account for dynamic changes in the population structure over the data collection period.

Despite these limitations, our findings have important implications for policy, clinical care, and future research. Our observations suggest that communally-oriented cultural features among AAs may protect against suicide among this ethnic group. Therefore, as the AA community in the US inevitably acculturates, suicide rates among this ethnic group may increase. Policymakers seeking to prevent suicide among this group might seek to promote community participation and civil societal growth among the AA community. Clinicians might pay particular attention to the mental health of AA patients, especially among those in highly acculturative atmospheres.

With regard to future research, this study is the first, of which we are aware, to consider suicide risk among Arab-Americans; more studies about suicidality among AAs in other spatial contexts, and among Arab populations in other non-Arab countries are needed. Moreover, studies about suicidal ideation and attempted suicide among AAs are in order. Also, researchers interested in ethnic differences in suicidality might explore mechanisms that explain differences in suicide risk between AAs and non-ethnic whites. Finally, investigators interested in the role of culture as a determinant of suicide might explicitly assess the relation between Arab cultural practice and suicide rates among AAs.
